# The value of lymphocyte count in determining the severity of COVID-19 and estimating the time for nucleic acid test results to turn negative

**DOI:** 10.17305/bjbms.2020.4868

**Published:** 2021-04

**Authors:** Yuanchao Li, Tuoyun Yang, Sicong Wang, Junbo Zheng, Jing Zhou, Min Jiang, Tong Zhou, Yang Cao, Hongliang Wang

**Affiliations:** 1 Department of Critical Care Medicine, the Second Affiliated Hospital of Harbin Medical University, Harbin, Heilongjiang, China; 2 Heilongjiang Province Medical Aid Group for CVOID-19, Wuhan, Hubei, China.; 3 Department of Critical Care Medicine, the Cancer Hospital of Harbin Medical University, Harbin, Heilongjiang, China

**Keywords:** COVID-19, lymphocyte count, SARS-CoV-2 nucleic acid test

## Abstract

Peripheral blood lymphocyte count is shown to be decreased in patients with COVID-19 in the early stage of the disease. The degree of lymphocyte count reduction is related to COVID-19 severity and could be used as an indicator to reflect the disease severity. Our aim was to investigate the value of lymphocyte count in determining COVID-19 severity and estimating the time for SARS-CoV-2 nucleic acid test results to turn negative. We retrospectively analyzed clinical data of 201 patients with severe and critical COVID-19. The patients were admitted to the West Campus of Union Hospital of Tongji Medical College of Huazhong University of Science and Technology. The data included age, gender, chronic disease, lymphocyte count, and SARS-CoV-2 nucleic acid test results. The age of patients in critically ill group was higher than in severely ill group (*p* = 0.019). The lymphocyte count of critically ill patients was lower than of severely ill patients. The cutoff value of lymphocyte count to distinguish between the critically ill and the severely ill was 0.735 × 10^9^/L (*p* = 0.001). The cutoff value of lymphocyte count for SARS-CoV-2 nucleic acid test results turning negative in severely and critically ill patients with chronic diseases (hypertension, diabetes, and coronary heart disease) was 0.835 × 10^9^/L (*p* = 0.017). The cutoff value of lymphocyte count for SARS-CoV-2 nucleic acid test results turning negative in severely and critically ill male patients was 0.835 × 10^9^/L (*p* < 0.0001). Lymphocyte count could be an effective indicator to predict COVID-19 severity. It may also be useful in determining the time for nucleic acid test results to turn negative in COVID-19 patients with underlying chronic diseases or male COVID-19 patients with severe and critical conditions.

## INTRODUCTION

COVID-19 caused by SARS-CoV-2 has been spreading globally since December 2019 [1,2]. The detection of SARS-CoV-2 nucleic acid by reverse transcription polymerase chain reaction (RT-PCR) is still the gold standard for the diagnosis of COVID-19. It is of great significance for determining the severity of the disease, discharge, and isolation [3]. However, clinical criteria that meet discharge standards are lacking. Specifically, it is still not possible to accurately determine when patients will test negative for SARS-CoV-2 during the course of the disease. Therefore, clinicians often measure this time based on the degree of clinical improvement in patients, however, this is subjective and lacks objective indicators.

The current studies have confirmed that peripheral blood lymphocyte count is decreased in patients with COVID-19 in the early stage of the disease. The degree of lymphocyte count reduction is related to the severity of the disease and could be used as an indicator to reflect the severity of the disease [4,5]. Simultaneously, the retrospective analysis of COVID-19 deaths has suggested risk factors affecting the prognosis of patients such as advanced age, male gender, and underlying diseases, especially hypertension [6-8].

Therefore, we aimed to explore lymphocyte count as an indicator of the severity of COVID-19 in patients, using SARS-CoV-2 nucleic acid testing results. To achieve this, we studied a total of 201 severely ill and critically ill COVID-19 patients admitted to the West Campus of Union Hospital of Tongji Medical College of Huazhong University of Science and Technology. We collected and analyzed their clinical data, including age, gender, chronic diseases, lymphocyte count, and SARS-CoV-2 nucleic acid detection results.

## MATERIALS AND METHODS

### Study participants

We conducted a retrospective study on 201 confirmed COVID-19 cases admitted to the intensive care unit (ICU) of the West Campus of Union Hospital affiliated with Tongji Medical College of Huazhong University of Science and Technology on January 29, 2020. Subjects, severely and critically ill, meeting the following criteria were included in this study: 1) age 18 years and older, 2) positive for novel coronavirus nucleic acid test, and 3) confirmed diagnosis of COVID-19 pneumonia. According to the Diagnosis and Treatment Plan of COVID-19 (7^th^ trial edition) of the National Health Commission, severely ill patients met the following criteria: 1) shortness of breath, RR ≥ 30 times/min, 2) oxygen saturation ≤93% at rest, and 3) arterial oxygen partial pressure/oxygen absorption concentration, PaO_2_/FiO_2_ ≤ 300 mmHg. The critically ill patients met the following criteria: 1) respiratory failure requiring mechanical ventilation, 2) shock, and 3) a combination of other organ failures requiring ICU care. Subjects meeting the following conditions were excluded from this study: 1) pregnant and lactating women and 2) those with diseases that may affect the efficacy or safety evaluation of this study (hematologic cancer, systemic lupus erythematosus, and rheumatoid arthritis).

### Ethics approval and consent to participate

Due to the special circumstances, this study was authorized by the Ethics Commission of Second Affiliated Hospital of Harbin Medical University (KY2020-007) and orally authorized by the Wuhan Union Hospital.

### Consent for publication

Written informed consent was waived by the Ethics Commission for emerging infectious diseases.

### Data collection

SARS-Cov-2 was detected by nucleic acid method using a nasopharynx swab at admission and during treatment of the enrolled patients and the corresponding absolute value of lymphocyte count before each nucleic acid detection was determined (LY#). We included patients who met the following criteria for discharge: 1) temperature returns to normal for more than three days, 2) respiratory symptoms improve significantly, 3) pulmonary imaging shows significant improvement in acute exudative lesions, and 4) nucleic acid test is negative for two consecutive times for samples of sputum, nasopharyngeal swabs, and other respiratory tract specimens, tested at intervals of at least 24 h. We collected lymphocyte counts from patients who met the above discharge criteria when their first nucleic acid test was negative [3].

### Statistical analysis

We employed IBM SPSS Statistics for Windows, Version 25.0. (IBM Corp., Armonk, NY) for statistical analysis in this study. Normally distributed data are expressed as the mean ± standard deviation. We applied the *t*-test for between-group comparisons of variables that showed normal distribution of data with homogeneous variances. Non-normally distributed data are represented by the median (upper quartile and lower quartile) and we used the non-parametric Mann–Whitney U test for between-group comparisons of non-normally distributed data or data with heterogeneous variance. The Chi-squared test was used for the comparison of counting data between groups. The Fisher’s exact test was applied when the theoretical value of comparison between two independent samples was less than 1. Binary logistic regression was used for multivariate impact analysis of severe and critical patients. A value of *p* < 0.05 was considered statistically significant.

## RESULTS

### Comparison of general data and lymphocyte count

Of the 201 cases included in this study, 167 cases were severe and 34 were critical. Table 1 compares the results of all indicators between these two groups. The age-range of patients in the critically ill group was significantly higher than that in the severely ill group (*p* = 0.019). The lymphocyte count of the critically ill group was significantly lower than that of the severely ill group (*p* = 0.001).

**TABLE 1:**
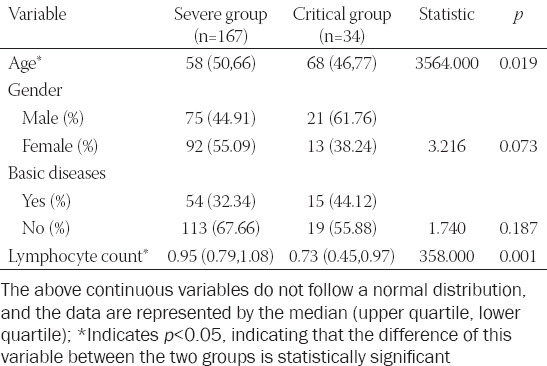
Comparison of data of severe and critical patients

Factors that were significantly different in the above analysis were used for stepwise binary logistic regression to identify factors that affect both severe and critical patients. The *p* values of the likelihood ratio test and the goodness of fit test of the model were less than 0.05, indicating that the model is statistically significant. T-lymphocyte count (*β* = 3.492; OR 0.030; *p* = 0.001) was found to have a significant effect on the severity of the disease, i.e., the lower the lymphocyte count, the more severe the disease.

**FIGURE 1:**
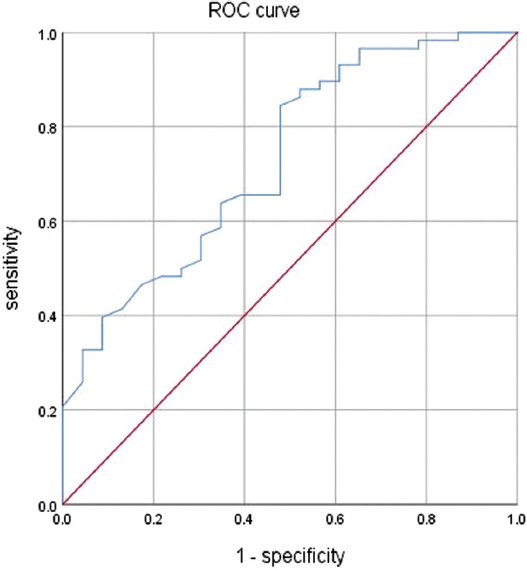
ROC curve of lymphocyte count in severe and critical patients.

Figure 1 displays the ROC curve for lymphocyte numbers with a statistical difference between the severely ill and the critically ill group. The area under the curve (AUC) value was 0.732, indicating that the lymphocyte number had a good diagnostic ability to distinguish the severely ill from the critically ill patients. The optimal diagnostic sensitivity was 0.845, and the specificity was 0.522. The cutoff value of lymphocyte numbers to distinguish between the severely ill and critically ill patients was 0.735 × 10^9^/L.

### Correlation between lymphocyte count and viral nucleic acid seroconversion from positive to negative

In this study, we included COVID-19 cases based on clinical disease severity (critical and severe), gender, presence of chronic diseases (hypertension, diabetes, and coronary heart disease), and age groups. We screened each group for patients who fulfilled the discharge criteria and had tested negative for SARS-CoV-2 twice consecutively by nucleic acid testing. We analyzed the correlation between SARS-CoV-2 positivity in each group of patients and their corresponding lymphocyte counts. The correlation between the first result and the corresponding lymphocyte count for the two consecutive negative tests in patients that met the discharge criteria are shown in Table 2.

**TABLE 2:**
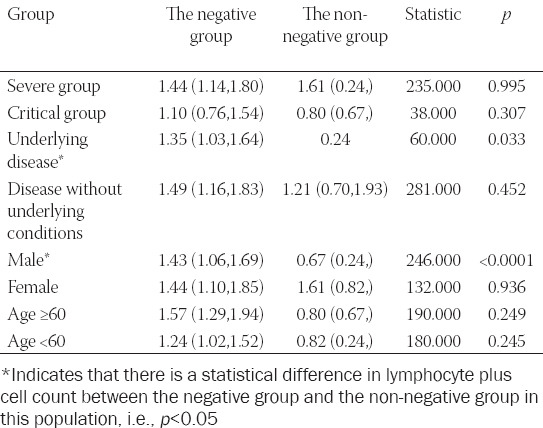
Results of comparison of lymphocyte counts of patients with and without viral conversion in each group

The severely ill group comprised 167 patients, including 163 patients who turned negative and four patients who did not. There was no significant difference in the lymphocyte counts between the negative group and the non-negative group (*p* = 0.995). Similarly, the critically ill group comprised 34 patients, including 19 patients who turned negative and 15 patients who did not. There was no significant difference in the lymphocyte counts between the negative group and the non-negative group (*p* = 0.307).

**FIGURE 2:**
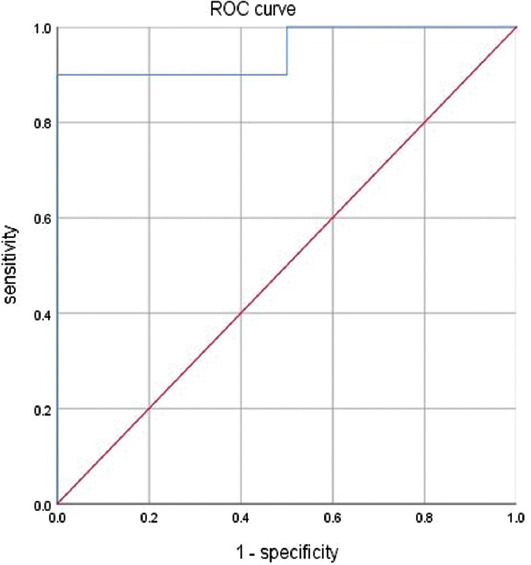
ROC curve of lymphocyte count in the group with underlying diseases.

The chronic disease group had 69 patients, including 61 patients who turned negative and 8 patients who did not. There was a statistical difference in the lymphocyte counts between the negative and the non-negative group (*p* = 0.017). Figure 2 displays the ROC curve for lymphocyte counts and the cutoff value that predicts negative testing for SARS-CoV-2. Its AUC value was 0.950, the optimal diagnostic sensitivity was 0.900, and the specificity was 1.000. The cutoff value of lymphocyte counts that could predict negative testing (seroconversion from positive to negative) was 0.835 × 10^9^/L. The non-chronic disease group comprised 132 patients, including 121 patients who turned negative and 11 patients who did not. There was no significant difference in the lymphocyte counts between the negative group and the non-negative group (*p* = 0.452).

**FIGURE 3:**
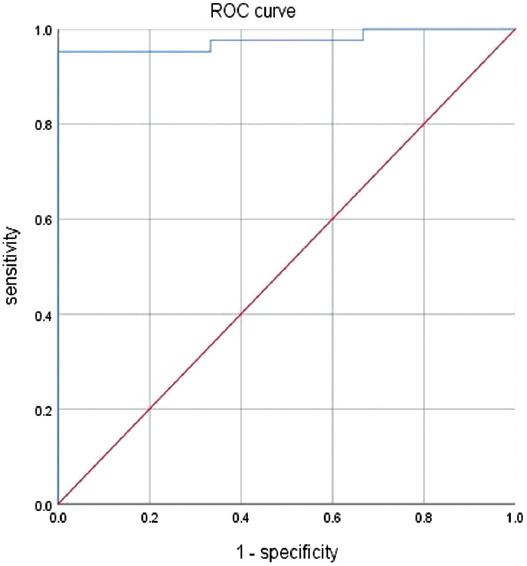
ROC curve for determining lymphocyte count that predicts seroconversion from positive to negative among males.

The male group had 96 cases, including 85 cases who tested negative and 11 cases who did not. There were statistically significant differences in the lymphocyte counts between the negative group and the non-negative group (*p* < 0.0001). Figure 3 shows the ROC curve for the lymphocyte count threshold that could predict a negative test result; this value was 0.835 × 10^9^/L. Its AUC value was 0.976, the optimal diagnostic sensitivity was 0.952, and the specificity was 1.000. The female group comprised 105 cases, including 97 patients who tested negative and 8 who did not (i.e., no conversion). There was no significant difference in the lymphocyte counts between the negative group and the non-negative group (*p* = 0.936).

There were 102 patients in the age group below 60 years, including 93 patients who tested negative and 9 who tested positive (i.e., negative for conversion). There was no significant difference in the lymphocyte counts between the negative group and the non-negative group (*p* = 0.249). There were 99 cases in the age group 60 years or older, which included 89 cases who tested negative (i.e., conversion from positive to negative) and 10 cases who continued to test positive (i.e., negative for conversion). There was no significant difference in the lymphocyte counts between the negative group and the non-negative group (*p* = 0.245).

## DISCUSSION

Our study revealed that COVID-19 patients in the critically ill group were older than those in the severely ill group. Also, their lymphocyte count was lower than that in the severely ill group. The negative results of the nucleic acid test in male or severe and critical patients with underlying chronic diseases, such as hypertension, diabetes and coronary heart disease, corresponded with the lymphocyte counts.

SARS-CoV-2 is a novel SARS-related coronavirus with high transmissibility with a basic reproductive number R0 ranging between 2.20 and 3.77 [9,10]. The *in vitro* culturing of this virus requires long incubation periods and can be carried out only in the highest biosafety level (P4) laboratories. Therefore, culturing the virus is of little significance for early detection, early reporting, and early treatment of the disease. In contrast, nucleic acid-based detection technology for this virus enables early diagnosis, high sensitivity, and high specificity [11]. Therefore, each edition of the Diagnosis and Treatment Plan for COVID-19 determines its own criteria for diagnosis and discharge on the basis of real-time fluorescent RT-PCR detection of SARS-CoV-2 nucleic acid in the respiratory tract or blood samples. Unlike SARS-CoV and MERS-CoV, the load of SARS-CoV-2 in respiratory specimens usually peaks in the 1^st^ week of onset before declining [12]. However, similar to SARS-CoV and MERS-CoV, the RNA load of SARS-CoV-2 in serum was closely correlated to disease severity and prognosis [13-15]. However, in the context of clinical treatment, there is no standard guideline to accurately determine the timing of SARS-CoV-2 nucleic acid detection. The clinical and radiographic manifestations of many patients improved significantly with time, but the actual viral load was still high. This also explains the presence of asymptomatic infection. The short interval of nucleic acid detection brings discomfort to patients during the detection process, and also increases the risk of infection of medical staff and utilizes limited medical resources, resulting in their wastage [16]. However, the long interval between tests, along with the prolonged length of hospital stay, undoubtedly, increases the risk of secondary infection. Currently, only a few studies have analyzed factors that influence the timing of seroconversion from positive to negative when nucleic acid-based testing is used. Hence, we aim to develop quantifiable standards to guide front-line clinical work.

Studies have confirmed that COVID-19 patients suffer an early-onset reduction of peripheral blood lymphocyte count [17,18], even lower than the normal reference range. Also, they found that the lymphocyte count in severely ill and critically ill patients was significantly lower than that seen in normal and mild patients [19]. However, further studies on whether there are differences in lymphocyte counts between severe and critically ill patients are lacking. Our study confirmed that lymphocyte count could be used as a reliable indicator to determine the severity of COVID-19 and prognosis in patients by dynamically observing the changes in lymphocyte count.

Lymphocytes play a central role in adaptive immunity as they are a core component of the immune system, responsible for antigen memory and recognition. Other coronaviruses, such as SARS-CoV, MERS-CoV and influenza viruses, also cause lymphocyte depletion with varying degrees in infected persons. This suggests that the counts of peripheral lymphocytes and their subsets significantly correlate with prognosis in critically ill patients [20,21]. The mechanism(s) underlying the reduction in lymphocyte count due to SARS-CoV infection have not yet been identified. It may be related to the following mechanisms. 1) Autopsy and pathological biopsy results confirmed a reduction in CD4+ and CD8+ T cells in the spleen and lymph nodes, suggesting local hemorrhage and necrosis. Furthermore, SARS-CoV-2 can directly invade immune organs, continuously proliferate, and infect more lymphocytes. 2) The cytotoxic effects of natural killer (NK) cells that have not been antigen sensitized in advance can kill the target cells as they act earlier [22] than T lymphocytes. In SARS-CoV-2 cases, NK cells may be consumed early as the virus attacks the immune system, leading to the failure of the replenishment of the cells, resulting in lower counts. This could also explain the existence of asymptomatic infections. 3) One of the important causes of immune inactivation in patients is the T cell depletion caused by increased expression of the inhibitory cytokine, interleukin 10 (IL-10), and the inhibitory molecules, programmed cell death protein 1 (PD-1) and T-cell immunoglobulin and mucin-domain containing-3 (TIM-3), on the cell surface and their subsequent inhibitory effects [23]. 4) Studies have also suggested that activation of the p53 signaling pathway may cause lymphocyte depletion in patients [24]. 5) Rapid replication of SARS-CoV-2 after infection leads to immune disorders in the early stage. It causes mass death of epithelial and endothelial cells and vascular leakage and activates a large number of pro-inflammatory cytokines and chemokines, including IL-2, IL-6, IL-7, IL-10, granulocyte colony-stimulating factor (G-CSF), interferon gamma-induced protein 10 (IP-10), monocyte chemoattractant protein 1 (MCP-1), macrophage inflammatory protein (MIP)-1a, and tumor necrosis factor alpha (TNFa). In other words, it leads to the so-called “cytokine storm.” Overexpression of TNFa can induce T cell apoptosis by binding to tumor necrosis factor receptor 1 (TNFR1) [17]. Therefore, a decrease in lymphocyte count may indicate limited replication of SARS-CoV-2, suggesting that lymphocyte counts can affect the outcome of nucleic acid-based detection methods that recognize viral RNA. This is supported by our observations on the change in lymphocyte count, which may be used to accurately determine the timing for SARS-CoV-2 testing when nucleic acid-based testing is employed.

Thus, lymphocyte count could be an effective indicator to determine the disease severity, length of stay, and prognosis of COVID-19 patients [25,26]. Our study confirmed that lymphocyte count could be used as a reliable indicator to determine the severity of COVID-19 and prognosis in patients by dynamically observing the changes in lymphocyte count. This is similar to the results reported by Tan et al., who retrospectively analyzed complete blood counts in cured and deceased patients during the time-course of the disease. Specifically, their study suggested that LYM% can be used as a reliable indicator to classify the moderate, severe, and critical ill patients independent of any other auxiliary indicators [25].

In addition, a number of studies confirmed age, gender, and underlying diseases, especially hypertension, to be closely related to the prognosis of COVID-19 patients [27,28]. Some studies found that the case fatality rate was as high as 14.8% in COVID-19 patients over 80 years of age, and that of males and females was 2.8% and 1.7%, respectively. The case fatality rate was as low as 0.9% in patients without underlying diseases. However, it was considerably high at 10.5%, 7.3%, and 6.0% in patients with previous cardiovascular diseases, diabetes, and hypertension, respectively. Therefore, scientists speculate that a normal functioning immune system of the patient, in the early stages of infection, may effectively suppress viral replication, helping the patient to transition to the recovery period faster. In patients with chronic diseases, such as hypertension, diabetes, and coronary heart disease, their immune system cannot control the rapid replication of SARS-CoV-2 and its attack on the immune system. Inflammation caused by SARS-CoV-2 leads to the downregulation of angiotensin-converting enzyme 2 (ACE2) and antibody-dependent enhancement (ADE) of host immune response to infection; both these processes eventually lead to patients developing the critical disease [29].

Based on the above hypothesis, we collected the relevant data from 201 patients whose disease course was classified as either severe or critical. We analyzed the value of lymphocyte counts in determining the severity of COVID-19 in patients and the timing of testing for SARS-CoV-2 when nucleic acid-based testing is used. We took into consideration various conditions, such as clinical types, gender, age, and the presence or absence of underlying chronic diseases. According to the meta-analysis of Zhao et al., a lymphocyte count of <1.5 × 10^9^/L may be useful in predicting the severity of clinical outcomes. Still, more studies that focus on lymphocyte changes in COVID-19 are needed to confirm the predictive ability of lymphopenia in COVID-19 [30]. Nevertheless, we show that a specific lymphocyte count of 0.735 × 10^9^/L can be used to predict the severity of the disease, which is an easier and more convenient method. Moreover, the lymphocyte count of critically ill patients was often lower than this. The viral RNA shedding time for shedding from the respiratory tract was significantly shorter in patients with normal B-cell counts on admission than in those with decreased B-cell counts [31], and patients with less active T-cell responses during the initial phase of infection shed viral RNA longer [32]. We found that when the lymphocyte count of such patients recovers to 0.835 × 10^9^/L, the nucleic acid test results are more likely to turn negative. Finally, we observed that the dynamic changes in lymphocyte count could improve the accuracy of nucleic acid-based detection and facilitate the assessment of patient condition and prognosis.

This study has three limitations. First, this is a retrospective study. The cases were from the critical disease area of Wuhan Union Hospital. We collected the samples from severely or critically ill patients. Second, there are currently only a few relevant studies available, and this study only explored the correlation between lymphocyte count and negative nucleic acid test results. Therefore, more laboratory test results need to be comprehensively analyzed in future studies to improve accuracy. Third, for greater convenience in statistical analysis, we used a range of lymphocyte count that did not account for the effects of age and gender on the lymphocyte count.

## CONCLUSION

Lymphocyte count could be an effective indicator to predict the severity of COVID-19 patients, and we show that dynamic changes in lymphocyte count in COVID-19 patients can be used to determine disease outcomes. Importantly, a lymphocyte count of 0.735 × 10^9^/L can be used to predict disease severity in patients, especially for determining the timing at which male patients with underlying chronic diseases or severe and critical conditions will seroconvert from positive to negative for SARS-CoV, thereby meeting discharge standards. When the lymphocyte count of such patients recovers to 0.835 × 10^9^/L, the nucleic acid test results are more likely to turn negative. Clinically, this information will help physicians guide patients in the timing for nucleic acid testing and improve the detection rate of negative results to meet discharge standards.
